# A Novel Sensor System for Measuring Wheel Loads of Vehicles on Highways

**DOI:** 10.3390/s8127671

**Published:** 2008-12-02

**Authors:** Wenbin Zhang, Chunguang Suo, Qi Wang

**Affiliations:** 1 Department of Automation of Testing and Control, Harbin Institute of Technology, Harbin, P.R. China; E-Mail: wangqi@hit.edu.cn; 2 MEMS Center, Harbin Institute of Technology, Harbin, P.R. China; E-Mail: suochungguang@126.com

**Keywords:** Weigh-In-Motion (WIM), Embedded concrete strain sensor, Vehicle wheel loads, Multiple sensors system

## Abstract

With the development of the highway transportation and business trade, vehicle Weigh-In-Motion (WIM) technology has become a key technology for measuring traffic loads. In this paper a novel WIM system based on monitoring of pavement strain responses in rigid pavement was investigated. In this WIM system multiple low cost, light weight, small volume and high accuracy embedded concrete strain sensors were used as WIM sensors to measure rigid pavement strain responses. In order to verify the feasibility of the method, a system prototype based on multiple sensors was designed and deployed on a relatively busy freeway. Field calibration and tests were performed with known two-axle truck wheel loads and the measurement errors were calculated based on the static weights measured with a static weighbridge. This enables the weights of other vehicles to be calculated from the calibration constant. Calibration and test results for individual sensors or three-sensor fusions are both provided. Repeatability, sources of error, and weight accuracy are discussed. Successful results showed that the proposed method was feasible and proven to have a high accuracy. Furthermore, a sample mean approach using multiple fused individual sensors could provide better performance compared to individual sensors.

## 1. Introduction

Accurate measurement of vehicle static axle or wheel loads has long been a major objective of highway engineers. The static weight of a vehicle is used to provide a basis for pavement analysis and design. Traditionally, these weights have been collected by pulling the vehicles off the roadway and weighing them at weigh stations while the vehicles are at rest. The static weighing of vehicles in highways has several disadvantages, including being time consuming, expensive, and dangerous on heavily travelled roads.

The concept of Weigh-In-Motion (WIM) was introduced more than fifty years ago [1]. WIM is the process by which the static weights of vehicles are determined by measuring wheel load while the vehicles are in motion. There are several advantages of weighing vehicles while they are in motion rather than at rest [2], which include savings in time and cost, and being safer to operate on busy roads.

Traditional highway WIM systems tended to use weighing devices set in the road in one lane [3]. Many such systems are still in use, including some developed in recent years. Wear and tear is severe, often resulting in a short lifespan. Unless the approach pavement is particularly smooth, these systems are often inaccurate because the devices are subject to large load fluctuations depending on vehicle speed, vehicle suspension characteristics and road roughness [4]. Many new technologies such as Multi-sensor WIM [7], Fiber-optic WIM [8-10] and Capacitive sensor WIM [11] were developed for improving accuracy and longer lifespan. Unfortunately, although the current WIM technologies offer many advantages, they still suffer from high installation and maintenance costs and low accuracy. Bridge-WIM [5-6, 20-21] could supply vehicle weight data rapidly and at a low cost, but it is hard to obtain separate axle weights as the bridge is under stress from the moment the vehicle enters the span.

A novel sensor system is proposed for measuring wheel loads of vehicles on highways. The proposed approach is based on the concept of monitoring strain response in the surface of rigid pavement affected by the moving vehicular loads. Multiple embedded concrete strain sensors are deployed in a freeway lane to directly measure the dynamic strain response of the pavement. The strain response is related to the instantaneous, dynamic wheel load on the pavement by the passage of vehicles in the vicinity of the strain sensor. The measurements can be processed to filter out noise and extract important features of the dynamic load. The relation between pavement response and the dynamic load can be obtained through calibration using a known wheel load vehicle. The article is organized by the following parts: Section 1 – Introduction; Section 2 – Description of the measurement method; Section 3 – Experimental test bench; Section 4 – Experimental results and analysis; Section 5 – Conclusion.

## 2. Description of the methodology

Current WIM systems are mainly based on three types of sensors: bending plate, piezoelectric and single load cells. All three sensors must be located in the path of the vehicle. The sensors register the vertical dynamic tire forces provided by the passing vehicles. Two sets of calculations must be performed on the WIM measurements to determine pavement loading under dynamic traffic conditions:

1. The measurements are used first to estimate the static weight of the axle. Accurate estimates of the axle load require continuing calibration of the WIM station.
2. The axle load estimates are then used to obtain the actual dynamic load that the pavement experiences. Calculations of the dynamic load depend on complex vehicle-pavement interaction models, which are hard to calibrate. Typically, these models are used to simulate dynamic loads.

The traditional measurement method just uses the interactions between the sensor and the vehicle's tires that make the measurement inaccurate because the sensor cannot cover the whole wheel path along the driving direction.

The proposed approach differs from the traditional one in two ways: 1) the sensing modality; 2) the computation of the dynamic wheel loads. The method is based on the principle that when a dynamic force is applied by a vehicle (via its tires), the amplitude of the tensile stress at the bottom of the surface layer increases as the force increases. However, the relation between tire force and displacement of pavement depends on the shape, size, and the structure of the rigid pavement. For numerical example, the rigid pavement system is modeled using model of a plate of infinite extent on a viscous Winkler foundation subjected to moving loads with amplitude variation to investigate stress and displacement response of rigid pavement [12]. With consideration of viscous damping, the maximum deflection and stress of pavement tend to decrease with increasing frequency. The velocity effect can be negligible for moving harmonic loads within the practical range of the vehicle velocities.

**Figure 1.**
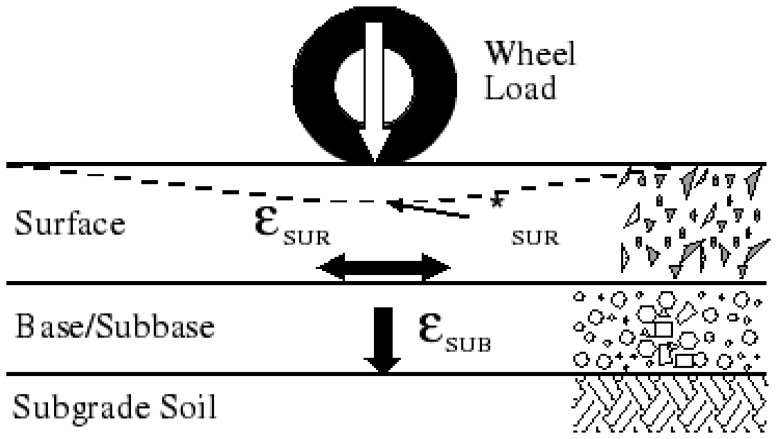
Illustration of the strain caused by moving wheel loads.

Actually, when the strain is not too large, rigid pavement behaves like a linear spring, which the displacement is proportional to the tires' force. In the linear range of rigid pavement response, the stress from vehicle tires is proportional to the strain (refer to Figure 1.), i.e. *ε_SUR_* = σ / *E*, where the longitudinal strain *ε_SUR_* is induced by the vehicle wheel load, σ is the stress and *E* is elastic constant, called the Young's modulus. In this research, the combination of embedded concrete strain sensors is used as WIM sensor.

**Figure 2.**
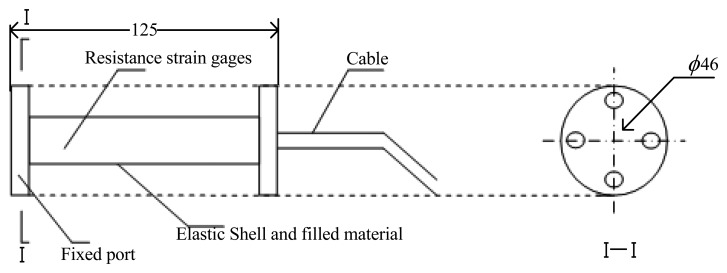
Structural sketch of the strain sensor (Units: mm).

**Figure 3.**
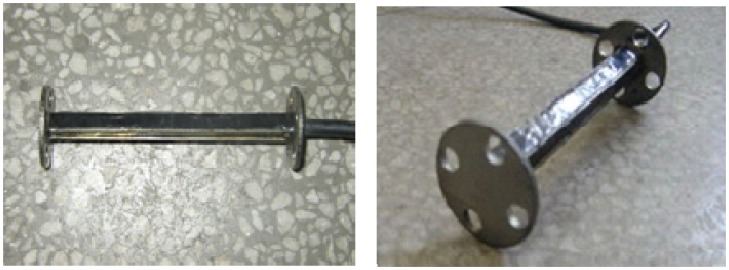
Photos of the strain sensor.

This paper is based on the use of a CMYB-YB-S125 embedded concrete strain sensor (designed by Qulang Information Ltd. Beijing of China) as WIM sensor. Figures 2 and 3 show the structural sketch of the embedded concrete dynamic strain sensor and the photos, respectively. The embedment strain sensors are designed for direct embedment in concrete pavement. The dimension of the strip strain sensors is 12.5 cm gauge length, 1.2 cm width and 1 cm thickness. It is an uniaxial embeddable strain gauge, with self-temperature-compensation, a resistance of 350 ± 0.5 Ω. The gauge factor is 2.0 and the modulus of elasticity is 30,000 Mpa. Its standard range is -1,500 *με* (compressive strain) and +400 *με* (tensile strain). The frequency range, which is used for measuring dynamic strain response in the concrete pavement subjected to moving vehicular loads, can extend from 0 Hz to 3 kHz. It is extra rugged to resist bending and has large flanges to provide greater engagement area.

**Figure 4.**
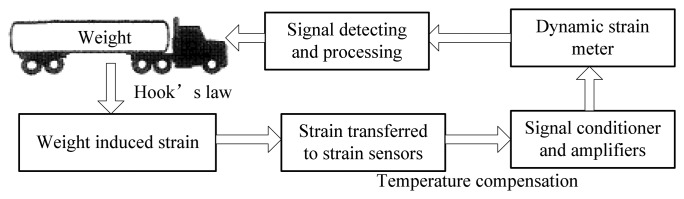
Flowchart of the proposed WIM system.

**Figure 5.**
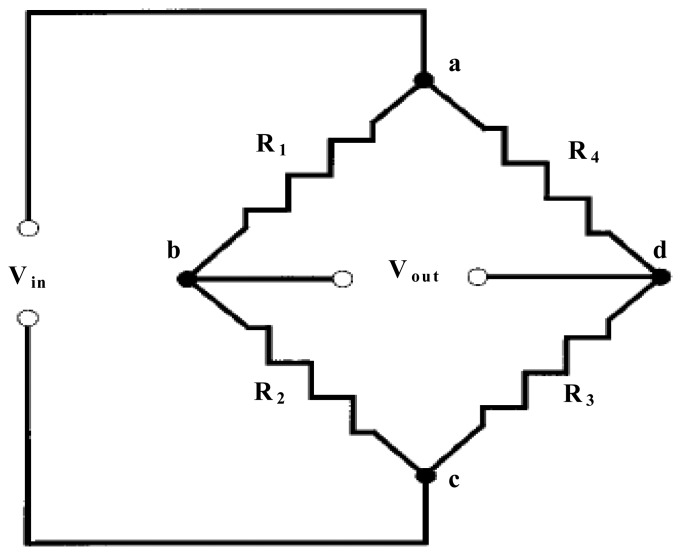
The Wheatstone bridge used to measure the relative resistance of strain gauges.

The basic principle of the proposed WIM system is illustrated in Figure 4. When a vehicle passes over the WIM sensor, the vehicle wheel loads will induce a deflection of the pavement and cause the strain of strain sensors in the longitudinal direction. Since the embedded concrete strain gauge is tightly epoxied together with the strain sensor, the piezoresistive material of the strain gauge will produce a relative resistance change. The relative resistance change of piezoresistive gauges is usually measured using a Wheatstone bridge (refer to Figure 5.). The bridge output, Equation 1, is zero when the balance condition, Equation 2, is met.

(1) $$V_{\text{out}}=V_{b}-V_{d}$$

(2) $$R_{1}R_{3}=R_{2}R_{4}$$

The balance condition is not longer meet if the resistance values undergo small changes see Figure 5. The Wheatstone bridge is used to measure the relative resistance of strain changes Δ*R*_1_, Δ*R*_2_, Δ*R*_3_, Δ*R*_4_. The higher order terms neglected, the results is shown in Equation 3 for the bridge output:

(3) $$V_{\text{out}}=V_{in}\frac{R_{1}R_{3}}{\left(R_{1}+R_{2})(R_{3}+R_{4}\right)}(-\frac{\Delta R_{1}}{R_{1}}+\frac{\Delta R_{2}}{R_{2}}-\frac{\Delta R_{3}}{R_{3}}+\frac{\Delta R_{4}}{R_{4}})$$

When *V_out_* is measured, the quantity of strain will be calculated from Equation 4.

(4) $$\varepsilon_{\text{sur}}=\frac{V_{\text{out}}K}{k_{0}}$$

where *ε_SUR_* is the strain of the surface of rigid pavement. *V_out_* is the output of the dynamic strain meter, *K* is the sensitivity of the strain of the strain meter (here 200 *με*/1.2 *V*). *k*_0_ is the sensitivity of the strain gauge (here *k*_0_ = 2).

The measurements are processed in accordance with the following statistical relationship:

(5) W=F(x,v)

where *x* is peak of the strain response; *v* is speed of vehicle; *W* is axle weight and *F* is the functional relationship that must be estimated.

## 3. Description of field test bench

In order to test the proposed WIM system based on rigid pavement strain response, an experimental test bench are designed and deployed at a freeway location. WIM accuracy in measuring static axle loads is affected by vehicle dynamics and noise. Road surface roughness excites vibrations of heavy vehicle which results in dynamic tire force fluctuations. These have typical Root Mean Square (RMS) amplitudes of 10-30% of the static wheel loads [13-14]. The dynamic tire forces result from vehicle motion in two distinct frequency ranges:

1.5 to 4.5 Hz: Sprung mass bounce and pitch vibration modes;
8 to 15 Hz: Unsprung mass bounce and roll, ‘load-sharing’ suspension pitch modes.

Various experimental and theoretical studies [13-14] have shown that the lower frequency sprung mass modes usually dominate the dynamic tire forces generated by heavy vehicle on highways, except for vehicles which have axle group suspensions (particularly of the walking-beam type) with poorly damped bogie pitching modes.

A WIM system with one force sensor uses a single sample of a wheel force time history as an estimate of the static wheel load. For such a system, assuming ‘perfectly accurate’ sensors, it can be shown that the expected standard deviation of the error in static load estimation for a particular wheel is the RMS dynamic tire force. Thus the accuracy of a single sensor WIM system is limited fundamentally by vehicle dynamics. One solution to this problem is to ensure that the dynamic loads are small by building a very smooth lead-up to the WIM site of up to 120 m in length [3]. However, the using of low cost the proposed WIM sensors provides the possibility of using two or more sensors along each wheel path in order to compensate for the effects of the dynamic forces in determination of the static axle loads.

### 3.1. Consideration of the Test Location

**Figure 6.**
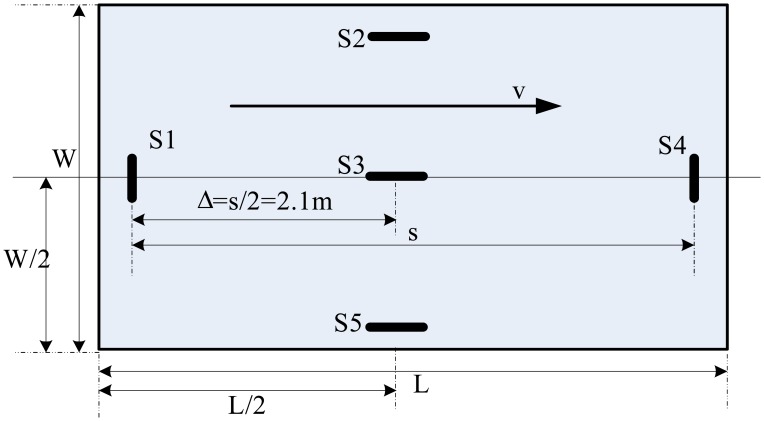
Deployment of proposed WIM system on a lane freeway.

Five embedded concrete strain sensors are deployed at the bottom of the pavement slab of a lane on freeway to measure the strain response of the pavement rather than to measure the forces of the axle load on the sensors as in current WIM stations (see Figure 6). S1, S3 and S4 are used as primary sensors to measure axle load, S2, S5 are used as adjunct sensors to determine relative location of wheel loads and monitor pavement structure. The reason for choosing sensors S1, S3 and S4 as WIM sensor is that its installation location can get better strain response depending on model analysis previously [12].

**Figure 7.**
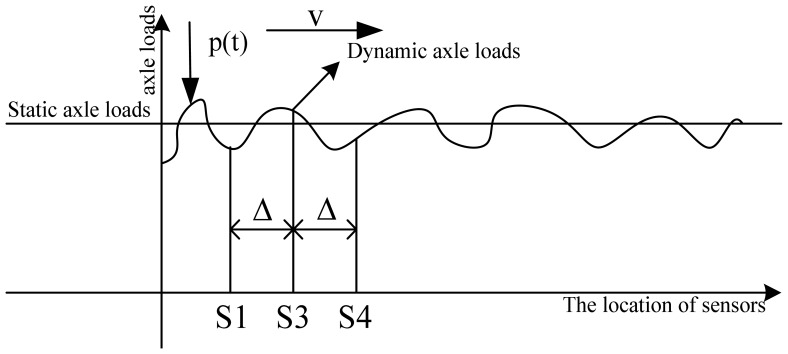
Cross-section of the proposed 3-sensor WIM array, traversed by dynamic tire force *p*(*t*) at speed *v*.

The installation of multiple-Sensor WIM (MS-WIM) can provide multiple measurements of the dynamic load of each axle. The use of MS-WIM revealed the importance of the known spatial repeatability in axle dynamics, defined as localized patterns in the dynamic loads applied to certain pavement locations, having magnitudes either higher or lower than their respective static values [15, 22]. Several algorithms were applied to MS-WIM systems for reducing errors due to dynamics and spatial repeatability, including the use of a maximum likelihood estimator, the use of signal reconstruction and the Kalman filtering method. The three algorithms were developed in the European WAVE project [16, 17]. Although these approaches showed theoretical promise, implementing them in practice yielded WIM accuracy no better than the one obtained from simple average-based calibration algorithms, due primarily to noise [18]. In this chapter, evenly spaced WIM arrays are examined. It is assumed that the outputs of the individual sensors are averaged to yield an estimate of the static loads. The simple average-based calibration algorithm was used to process three sensors data. Figure 7 gives illustration of cross-section of the proposed 3-sensor WIM array. In Section 4, measurements from the wheel load measuring bench will be used to examine the validity of multi-sensor WIM here.

### 3.2. The Description of the Instrumental Pavement

The site chosen for experimental the installation was on a section of test pavement at k 220+300 of Tong-Three state road, in Jiamusi of Heilongjiang province, Northeast China. Pavement cross sections and material properties is known.

The instrumental rigid pavement including five embedded concrete strain sensors located symmetrically below the surface of the pavement slab (refer to Figure 6) are used in the WIM measurement bench. Pavement slab width W=4.50 m; L=4.6 m is the length of the pavement; *v* is the direction of traffic movement; sensors S1, S2, S4, S5 are 25 cm under the edge of pavement; *s*=4.2 m is the distance between S1 and S4. This layout can be used to measure all the vehicles on the right side of the road [23]. Corresponding vehicle parameters can be extracted from multiple sensors (these will be introduced in Section 3.4).

### 3.3. Data Collection devices

**Figure 9.**
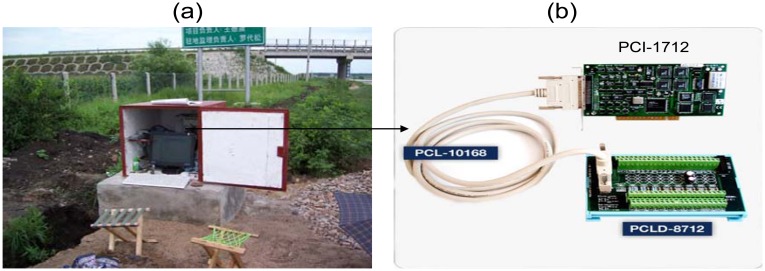
(a) Photo of the data collection device; (b) Data collection card and interface board.

For continuous acquisition of sensor data, data acquisition software and hardware is necessary. These systems are built around an industrial computer with a Windows operating system. This computer has a PCI-data acquisition board supporting 12 bit 16 single-ended or 8 differential analog inputs maximum up to 1 MHz sampling rate (Advantech Co. Ltd PCI-1712 Multifunction DAQ). The data acquisition system (see Figure 9b) is connected to the junction box of each section to record the dynamic pavement response resulting from a moving wheel load. The acquisition program, written in C++, provided for different sampling rates and times. A sampling rate of 2 kHz for 2 sec for each channel was found to be most appropriate for this installation.

### 3.4. Sensors' Response to Pavement Deflection

**Figure 10.**
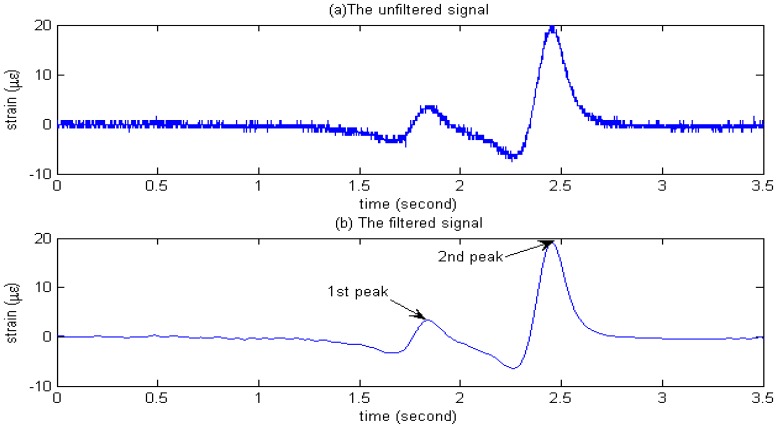
(a) Unfiltered and (b) Filtered strain time histories for moving two-axle vehicle.

After the installation, all of sensors are connected to a dynamic strain meter and the outputs of the dynamic strain meter are fed to the data acquisition system for data storage and processing. The output signal from the sensor as a two-axle vehicle passes over the instrument pavement is shown in Figure 10a. The elastic strain of sensors just happens and then the stress vanishes. To calculate the weight of vehicle, it is necessary to determine the peaks when vehicle passes over each of the strain sensors. These values correspond to the two predominant positive peaks in the strain time histories. To locate these local maxima, a Matlab program which searches for and returns all peaks associated with axle loads was developed. Ideally, only one maxima per axle will be identified; however, other maxima associated with noise are also present (refer to Figure 10a). From Figure 10a it can be seen that original signal is affected by noise and is difficult to process. To remove these unwanted peaks, it is important for signal de-noising to retain the accurate characteristics of the original signal. Recently, the wavelet transform, a scale-frequency representation of a signal, has become very popular tool in signal processing. This transform constitutes a sort of remedy for limitation involved in the Short Time Fourier Transform resolution, and it is usually applied to detection, extraction, compression and de-noising signals. The basic idea behind signal processing with wavelets is that, like in Fourier analysis, a signal can be decomposed into its component elements through the use of basis functions. In the case of Fourier, the basis functions are sine and cosine waves. In the case of wavelet analysis, the basis functions consist of the wavelet scale function and scaled and shifted versions of the mother wavelet function. In this paper, the signal is brought to a level 5 through the wavelet decomposition. The signal reconstruction is based on both the original approximation components and the detailed components modified by the soft threshold operation. The threshold selection rule was based on Unbiased Estimate of Risk (quadratic loss function) [19]. The mother wavelet function is sym8, which belongs to the symlets series. The result is shown in Figure10b, which demonstrates the feasibility and applicability of the de-noising method. The system measures the peak voltage of de-noising curve produced by the two axle of vehicle as the elastic strain, calculates the axle weights and sums them to obtain the gross weight.

**Figure 11.**
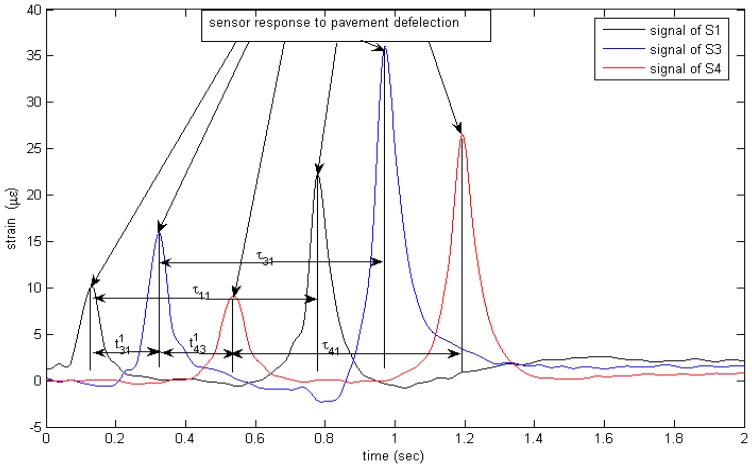
An example of sensor response to pavement deflection subjected to moving wheel loads.

Once the maxima corresponding to the front axle and the rear axle crossing the strain sensors have been determined, the speed can be calculated by dividing the distance between the strain sensor S1 and S3 (2.1 m) by the change in time between the axle first at S1 and then S3 (refer to Figure 11). Other vehicle parameters such as wheelbase can be calculated from the following equation: *WB* = *speed*/*τ*, where *τ* is time interval of adjacent peaks in the strain time histories. The number of peaks in the strain time histories is the number of axles. These parameters can be used to classify vehicles.

**Figure 12.**
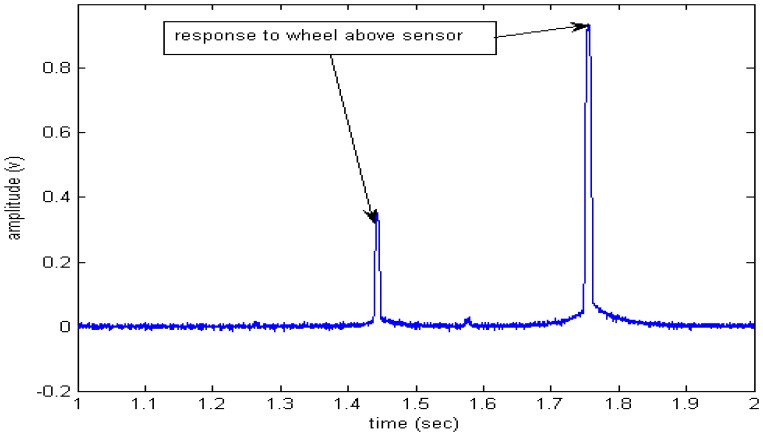
A piezoelectric sensor response directly measure moving tire force.

Compared with current piezoelectric WIM systems, it is obvious that signal duration from the proposed system is longer than current piezoelectric sensor (refer to Figures 11-12.). Because the pavement strain is the contribution of the whole wheel load to the pavement, it is not like the interaction between the tire and the sensor that just covers a little part of the force due to the narrow width of the sensor compared with the contact patch of the tire, so higher measurement accuracy is expected by considering the pavement strain response.

In order to verify this analysis previously mentioned the measurements results are provided as follows.

## 4. Experimental Results and Analysis

In order to evaluate the proposed WIM system, calibration of method, the accuracy, error sources, experimental results from individual sensor and multiple sensors are discussed in this section.

### 4.1. Accuracy of WIM System

Usually, WIM systems are used to estimate vehicles' static weight from the measurement of dynamic weights. The difference between static and dynamic weight is considered to be WIM error.

To setup a criterion to describe WIM system's performance, precision errors and accuracy errors are given out.

The WIM accuracy is represented as follows:

(6) $$A=\frac{W_{d}-W_{s}}{W_{s}}\times 100\%$$

where *A* is WIM measurement accuracy; *W_d_* is axle weight or gross weight measured by the WIM system; *W_s_* is axle weight or gross weight measured by a static scale.

A WIM system is defined to be accurate if the mean value of equation (6) for a sample of weight observations does not differ significantly from zero. The bias from that mean value is considered to be a systematic error existing in the WIM measurement. Proper calibration of a WIM system can minimize systematic error by choosing a sample of vehicles from the traffic stream that is representative of the vehicles intended to weigh. Considering the ‘Accuracy’ in equation (6) as a statistic variable, the systematic error can be defined as:

(7) $$\mu_{A}=E[A_{n}]$$

Where *μ_A_* is the systematic error; *A_n_* is the variable defined by equation (6), and the *n* subscript represents the number of samples.

Based on the systematic error's definition, the statistical precision given in (7) can be defined as the range within which a specific percentage of all observations can be expected to fall, which is represented as follows:

(8) $$\mu_{A}\pm X_{\alpha /2}*\sigma_{A}$$

where *μ_A_* is a defined in (7); *X_α_*_/2_ is the critical value from the standard normal distribution associate with the level of confidence *α*; *σ_A_* is the standard deviation of *A.*

### 4.2. Field Calibration Using Vehicle Loads

In this section a devised field sensor system calibration method is described. The method requires a vehicle with known static loads to be driven over the sensor array a number of times at low speeds, so as to minimize the dynamic loads. A program was written in C++ to read-in a number of processed data files associated with these tests and to calculate average calibration factors for each sensor using the static loads from one or more of the axles.

**Figure 13.**
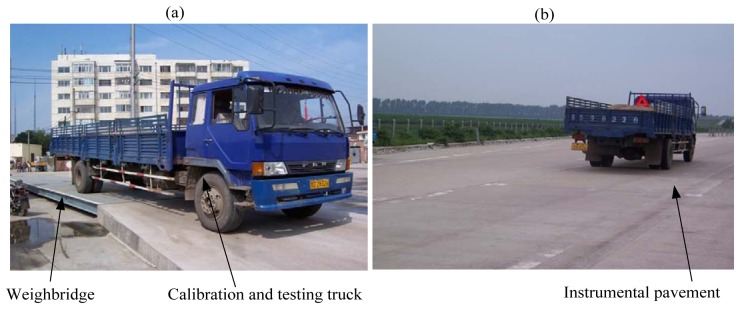
A two-axle truck for calibration and testing (a) vehicle static wheel load on a static whole-vehicle weighbridge; (b) vehicle passing the instrumental pavement

A two-axle truck was used for calibration and testing on the proposed WIM system (refer to Figure 13). The vehicle was arranged into a tandem axle combination. The combination was coded A1 and A2 and static axle weight was described in Table 1. The vehicle combination was selected to be relatively representative of a typical Chinese truck.

Each vehicle static wheel load was weighed on a static whole-vehicle weighbridge of approximately 15 m length immediately prior to, or after the testing. The weighing procedure involved driving the vehicle on and then off the weighbridge, one axle at a time, and recording the weight of each axle combination. This enabled two estimates of the static load of each axle to be obtained as well as the gross weight of the vehicle. The individual static axle loads are provided in Table 1.

Each vehicle load combination was driven over the proposed WIM system at nominal speed of 5, 10, 20, 30, 40, 50 km/h in a forward direction over the test bench. In a few cases, a speed of 60 km/h was achieved. At least ten repetitions were performed at each test condition and the matrix of tests is summarized in Table 1. A total of 420 test runs was performed on the two-axle vehicle during four days of testing.

### 4.3. Experimental Results from Individual Sensor and Multiple Sensors

#### 4.3.1. Experimental results from individual sensor

**Figure 14.**
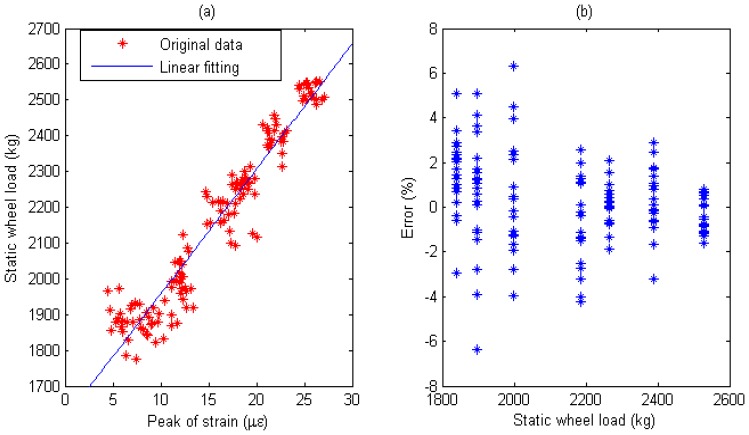
(a) Weight calibration function (y=36.6x+1575) for S1 on drive axle. (b) Error of axle weight measurement for S1 on drive axle.

**Figure 15.**
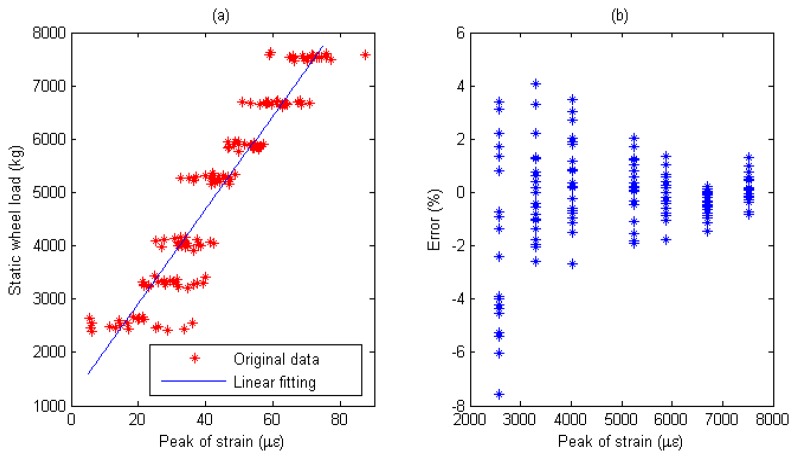
(a) Weight calibration function (y=97.9x+660.4) for S1 on trailer axle. (b) Error of axle weight measurement for S1 on trailer axle.

**Figure 16.**
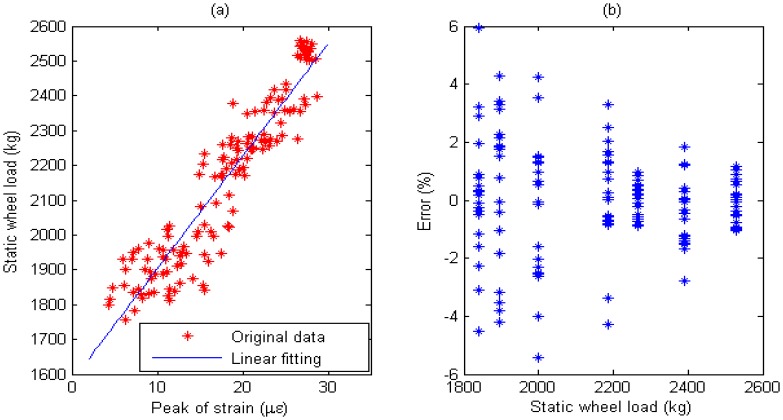
(a) Weight calibration function (y=31.2x+1599) for S3 on drive axle. (b) Error of axle weight measurement for S3 on drive axle.

**Figure 17.**
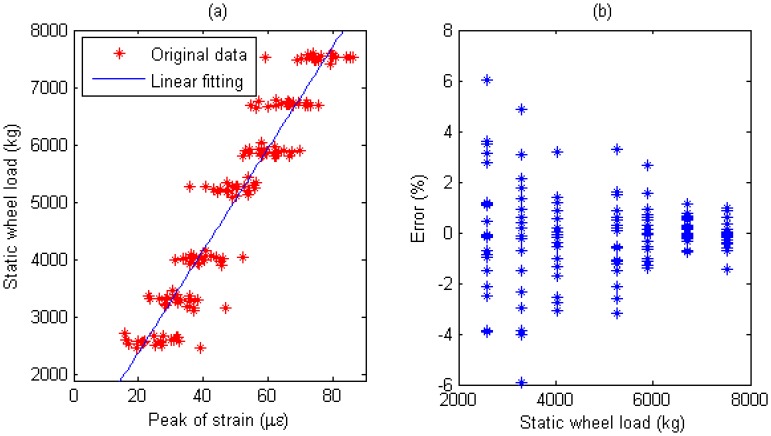
(a) Weight calibration function (y=88.8x+614.4) for S3 on trailer axle. (b) Error of axle weight measurement for S3 on trailer axle.

**Figure 18.**
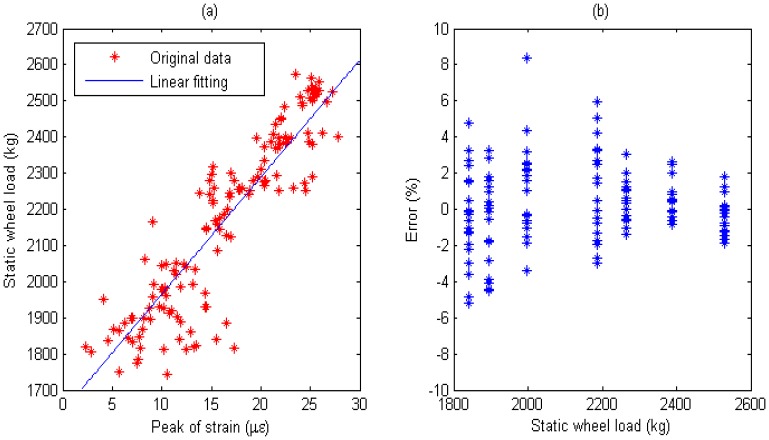
(a) Weight calibration function (y=32.6x+1640.2) for S4 on drive axle. (b) Error of axle weight measurement for S4 on drive axle.

**Figure 19.**
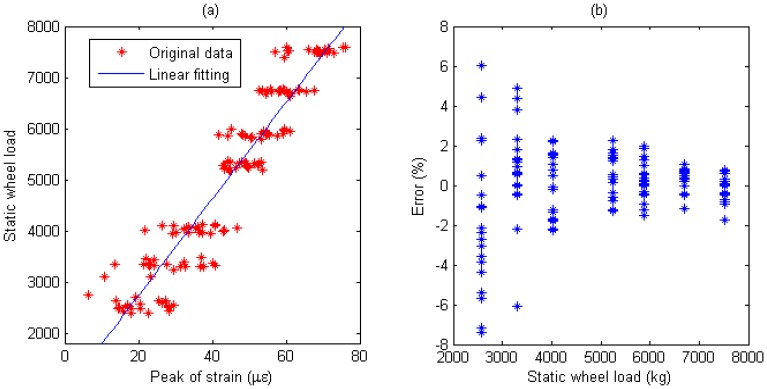
(a) Weight calibration function (y=94.4x+843.7) for S4 on trailer axle. (b) Error of axle weight measurement for S4 on Trailer axle.

Multiple calibration constants of each WIM sensor are calculated (refer to Figures 14, 15, 16, 17, 18 and 19a). In this procedure the calibration constant for the drive axle and trailer axle are different. Applying the linear algorithm, i.e., multiplying a calibration constant and the sensor output, then using the static weight of the truck as standard, the measurement error can be calculated by the following equation:

(9) $$\text{error}=\frac{K\;\varepsilon_{i}-W_{s}}{W_{s}}\times 100\%$$

where *W_s_* is the static weight of a wheel; *K* is a calibration constant and *ε_i_* is the output of the *i*th strain sensor.

The measurement error for individual WIM sensors are shown in Figures 14, 15, 16, 17, 18 and 19b, respectively. The nonlinear pavement response can be explained by the differences in calibration constants between the drive axle and trailer axle. The lower calibration constant of the drive axle than the trailer axle (refer to Figures 14, 15, 16, 17, 18 and 19) indicates the pavement reacts more stiffly to light wheel loads. The data are irregular and sparse for the drive axle and the measurement error is bigger than the trailer axle.

Using simple sample average method, the measures of WIM system with arrays with 3 evenly-spaced sensors (refer to Figure 7.) are defined as Equation (10). The relative error *A_f_* for test run *i* is defined as Equation (11):

(10) $$W_{f}=\frac{W_{s1}+W_{s2}+W_{s3}}{3}$$

(11) $$A_{f}=\frac{W_{f}-W_{s}}{W_{s}}\times 100\%$$

where *W_f_* and *W_s_* are the estimated (in-motion) by three sensors array and the reference (static) weights respectively. We use confidence interval to represent the statistics of the proposed system performance. The confidence is:

(12) $$C=\overset{d_{e}}{\;}/\underset{n}{\;}$$

where *n* is the total number of tests; *d_e_* is the number belonging to error interval [-*e*, +*e*].

In this paper, with the same speed and different axle loads, the total number of the calibration vehicle passing over the sensors is 70 times. We can get the value of *d_e_* and the confidence level *C* for each individual of sensors and three sensors array.

S1: For Drive axle when error interval ∈ [-6%, +6%],*d_e_* = 65, *C*=0.928; For Trailer axle when error interval ∈ [-6%, +6%], *d_e_* =66, *C*=0.943; (refer to Figure 14 (b) 15 (b))

S3: For Drive axle when error interval ∈ [-6%, +6%], *d_e_* = 67, *C*=0.957; For Trailer axle when error interval ∈ [-6%, +6%], *d_e_* =67, *C*=0.957; (refer to Figure 16 (b) 17 (b))

S4: For Drive axle when error interval ∈ [-6%, +6%], *d_e_* = 66, *C*=0.943; For Trailer axle when error interval ∈ [-6%, +6%], *d_e_* =66, *C*=0.943; (refer to Figure 18 (b) 19 (b))

**Figure 20.**
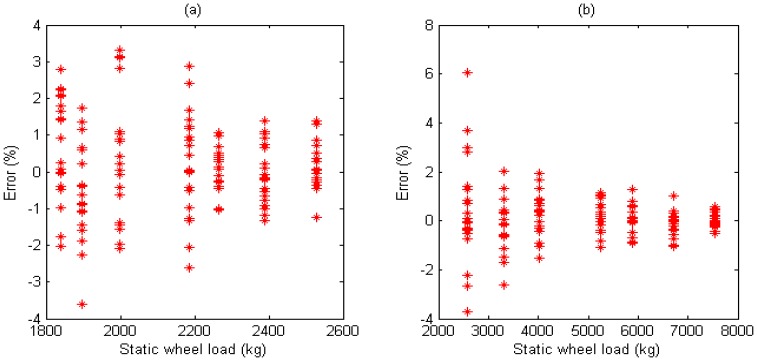
(a) Error of weight measurement for fusion three sensors on drive axle. (b) Error of weight measurement for fusion three sensors on trailer axle.

Sensors array: For Drive axle when error interval ∈ [-4%, +4%], *d_e_* = 70, *C*=1.000; For Trailer axle when error interval ∈ [-4%, +4%], *d_e_* =69, *C*=0.986; (refer to Figure 20 (a), (b))

Compared with individual sensor, three sensors array fusion increased confidence level and provided higher accuracy.

### 4.4. Repeatability of System

Short-term repeatability of results are show in calibration curve (refer to Figures 14a, 15, 16, 17, 18 and 19a). Sensor data points at similar speeds, wheel loads and passing location were acquired at times of up to 1 hour or more part, each run taking some 2 hr in all. The standard deviation of the strain difference of data points from the curve of linear fit to the data in this case was 3.3% of the maximum ordinate. Ten experimental results under the same loading conditions are plotted in Figures 14a, 15, 16, 17, 18 and 19a demonstrating that the proposed system has good repeatability.

### 4.5. Sources of Error

The major source of error in vehicle load determination was nonlinear pavement response and the speed of vehicle. Other than nonlinearity and errors due to lack of knowledge of the real no load datum applicable to a particular wheel pass, other sources of error include wheel position determination, different pavement responses at different vehicle speeds, unequal load distribution to wheels of an axle due to unsymmetrical load placement and to road camber, differences of pavement response to tire contact width, length and pressure, differences in spacing of dual wheels and unequal tire loads in dual wheels. Some of these could be accommodated by further instrumentation, such as more transducers in the right hand wheel path, designed to determine wheel width and wheel position. Generally, the error due to the foregoing factors, other than nonlinear response and the speed of vehicle, is believed to be individually minor.

## 5. Conclusions

In a WIM system, the gross weight or the axle weight of the passing vehicle can be measured dynamically by the sensors installed in or on the pavement. Traditional measurement method just uses the interactions between the sensor and the vehicle's tires that make the measurement inaccurate because the sensor cannot cover the whole tire patch along the driving direction. A novel WIM method based on pavement strain response is proposed in this paper. Since the pavement strain is caused by an entire moving vehicular wheel load; it covers a longer force duration time than current WIM methods, so higher measurement accuracy is expected by considering the pavement strain. The proposed method has more durability, lower system maintenance costs (due to its embedded installation), good concealment (improved road safety), little environmental impact (uninterrupted continuous measurement), low cost for individual sensors (providing the possibility of using multiple sensors in a WIM system). A major contribution of this paper was to design, build, and test of a novel experimental WIM system prototype based on a radically different approach than current WIM systems. The field tests and calibration experiments were presented. The results verified that using the embedded strain sensors was feasible and multiple sensors fusion could get higher accuracy and confidence level than individual sensor. In the paper, only experiments at the same nominal speed have been researched and analyzed and the accuracy of WIM measured vehicle weights has not been affected obviously by speed. In the future, experiments under high speed conditions could be performed and the effect of speed studied. Other features such as temperature properties of the sensor and installation issues are to be studied too. Furthermore, only the sample mean multiple fusion method has been research and tested in this paper, so in order to achieve the higher precision more algorithms are to be applied to the MS-WIM.

## Figures and Tables

**Figure 8.**
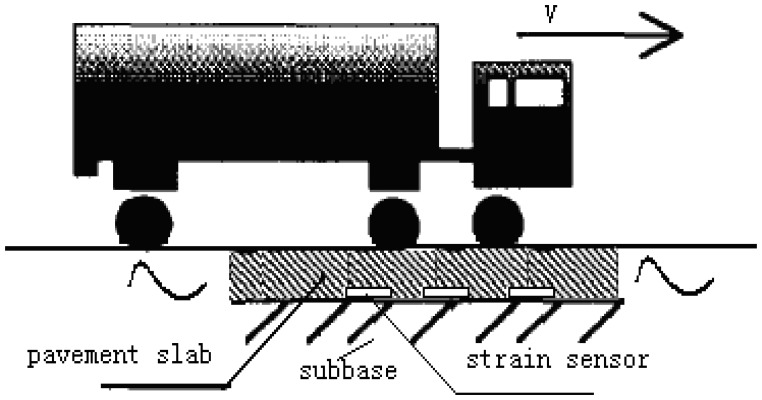
Illustration of the 3-axle trailer truck passing over the instrumental pavement slab.

**Table 1.** Static weight in field test.

**Number of Tests**	**Drive axle static weight (kg)**	**Trailer axle static weight (kg)**	**Axle group gross weight (kg)**	**Nominal speed (km/h)**

60	3680	5160	8820	5, 10, 20, 30, 40, 50
60	3790	6610	10440	5, 10, 20, 30, 40, 50
60	3990	8060	12070	5, 10, 20, 30, 40, 50
60	4380	10510	14870	5, 10, 20, 30, 40, 50
60	4540	11760	16330	5, 10, 20, 30, 40, 50
60	4780	13410	18170	5, 10, 20, 30, 40, 50
60	5060	15060	20100	5, 10, 20, 30, 40, 50
